# Functional analysis of the promoter of an early zygotic gene KLC2 in *Aedes aegypti*

**DOI:** 10.1186/s13071-018-3210-0

**Published:** 2018-12-24

**Authors:** Wanqi Hu, Zhijian Jake Tu

**Affiliations:** 1 Department of Biochemistry, Virginia Tech, 303 Fralin, Blacksburg, VA 24061 USA; 2 Fralin Life Science Institute, Virginia Tech, Blacksburg, VA 24061 USA

**Keywords:** Early embryo, male, development, gene drive, vector, infectious disease

## Abstract

**Background:**

*Aedes aegypti* is an important mosquito vector that transmits arboviruses that cause devastating diseases including Zika, dengue fever, yellow fever and chikungunya. Improved understanding of gene regulation in the early development of *Ae. aegypti* will facilitate genetic studies and help the development of novel control strategies of this important disease vector.

**Results:**

In this study, we demonstrated through transgenic assays that the promoter of an endogenous early zygotic gene KLC2 could drive gene expression in the syncytial blastoderm and early cellular blastoderm, which is a stage that the developing germline and the rest of embryo are accessible to genetic manipulation. An unexpected expression of the reporter gene in transgenic male testes was also observed. Further analysis confirmed the expression of the endogenous KLC2 in the testes, which was not detected in the previous RNA sequencing data.

**Conclusions:**

Our finding provided a new promoter element that can be used in future genetic studies and applications in *Ae. aegypti*. Moreover, our transgenic reporter assays showed that cautions are needed when interpreting RNA sequencing data as transient or tissue-specific transcription may go undetected by RNAseq.

**Electronic supplementary material:**

The online version of this article (10.1186/s13071-018-3210-0) contains supplementary material, which is available to authorized users.

## Background

*Aedes aegypti* is a major vector for Zika, dengue fever, yellow fever and chikungunya. These diseases affect tropical and subtropical regions worldwide, including Africa, the Americas, the Eastern Mediterranean, Southeast Asia and the Western Pacific. The rapid spread of Zika and the resulting microcephaly has caused major devastation in recent years. In the 2015 outbreak in Brazil, an estimated 440000-1300000 cases of Zika virus infection and over 4700 suspected cases of microcephaly have been reported [1, 2]. The global incidence of dengue has increased dramatically in recent decades and nearly half of the world population is now at risk [3]. There are a few ongoing trials for vaccines. However, no specific treatment for dengue exists and current prevention depends solely on effective vector control [3], which is hindered by increasing insecticide-resistance [4–7]. Novel control strategies, informed by improved understanding of mosquito biology, are still needed. Population suppression and population replacement are two such strategies, where genetic-modified mosquitoes are released to decrease the number or ability of mosquitoes transmitting diseases [8–13]. These methods require genetic manipulations and will benefit from precise control of transgene expression. For example, ectopic expression or knockdown of genes involved in sex-determination during early embryonic stages could produce genetic sexing strains, which are useful for population suppression approaches like the sterile insect technique [14–16]. To this end, a thorough knowledge of mosquito biology and gene regulatory elements is necessary.

Despite the great burden *Ae. aegypti* puts on public health, only a limited number of its promoter sequences have been described and tested in genomic context. Several promoters derived from endogenous genes are functional in midgut, fat body, salivary gland, ovary or testes [17–22]. Akbari and colleagues [23] reported 4 promoters which can drive strong maternal germline specific expression and be used for building a gene drive system termed MEDEA (Maternal Effect Dominate Embryonic Arrest [24]). No endogenous promoter that drives early zygotic gene expression has been tested *in vivo* in *Ae. aegypti*. Such a promoter is another major component of the MEDEA drive. In addition, early zygotic expression in the syncytial blastoderm and early cellular blastoderm affords the transgene or other factors access to the developing germline and the rest of embryo, which is beneficial to genetic manipulation. We previously sequenced staged *Ae. aegypti* embryos and found a list of genes that expressed at the onset of the syncytial blastoderm stage. We demonstrated that the 1 kb promoter region of one of the early zygotic gene (EZG) KLC2.1 was able to drive gene expression in early embryos through transient reporter assays [25]. In this study, we took a further step to establish the promoter activity in transgenic mosquitoes and showed that the 1 kb region of the KLC2.1 promoter initiate transgene expression very early in the embryos of *Ae. aegypti*. We also observed an unexpected transgene expression in testes and confirmed the same expression of the endogenous gene KLC2.1. Our study provides the first functional early zygotic promoter for future studies in genetic-modified mosquitoes or gene functions. Moreover, our results highlight an example in which tissue-specific expression of a gene may be missed in even relatively broadly sampled RNAseq data. In the most recent *Ae. aegypti* L5 genome assembly [26], KLC2.1 is merged with KLC2.2 and we will refer to this gene KLC2 hereafter.

## Results

### Generation and characterization of transgenic mosquitoes bearing the KLC2 promoter

**Fig. 1:**
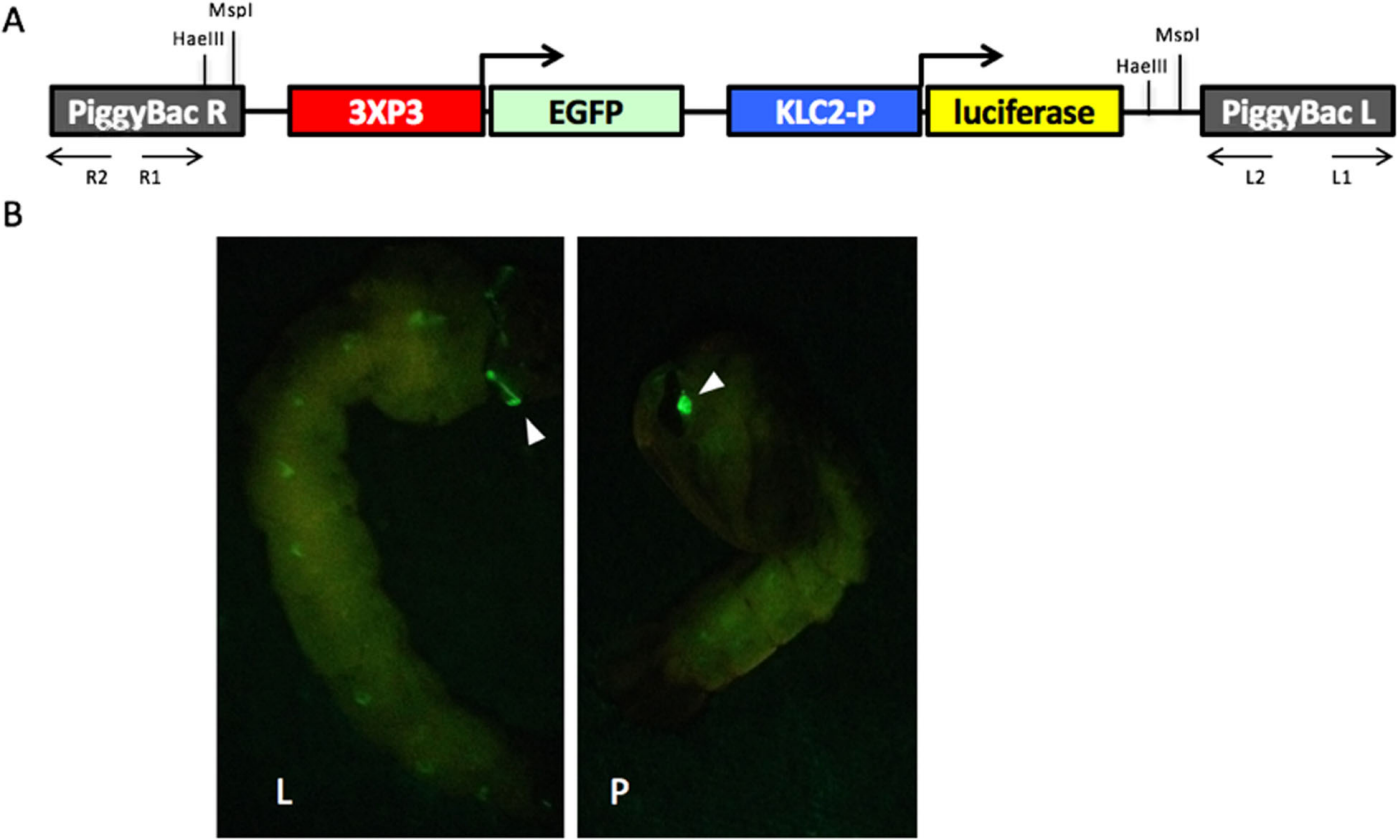
The structure of donor plasmid used in embryo injection (**a**) and expression of the EGFP marker in transgenic mosquitoes (**b**). **a** The donor plasmid contains a transformation marker, EGFP driven by the 3xP3 promoter, and a firefly luciferase coding cassette under the control of the 1kb upstream promoter sequence of KLC2 (KLC2-P). The entire sequence of the luciferase reporter cassette is provided as Additional file 6. *piggyBac* arms are also shown. Arrows indicate primer sets for inverse PCR (iPCR). Due to space limitation, nested iPCR primers are not drawn. All primers are shown in Additional file 3. **b** Images of transgenic positive larva (L) and pupa (P). Arrow heads point to EGFP marker expression in the eyes

**Fig. 2:**
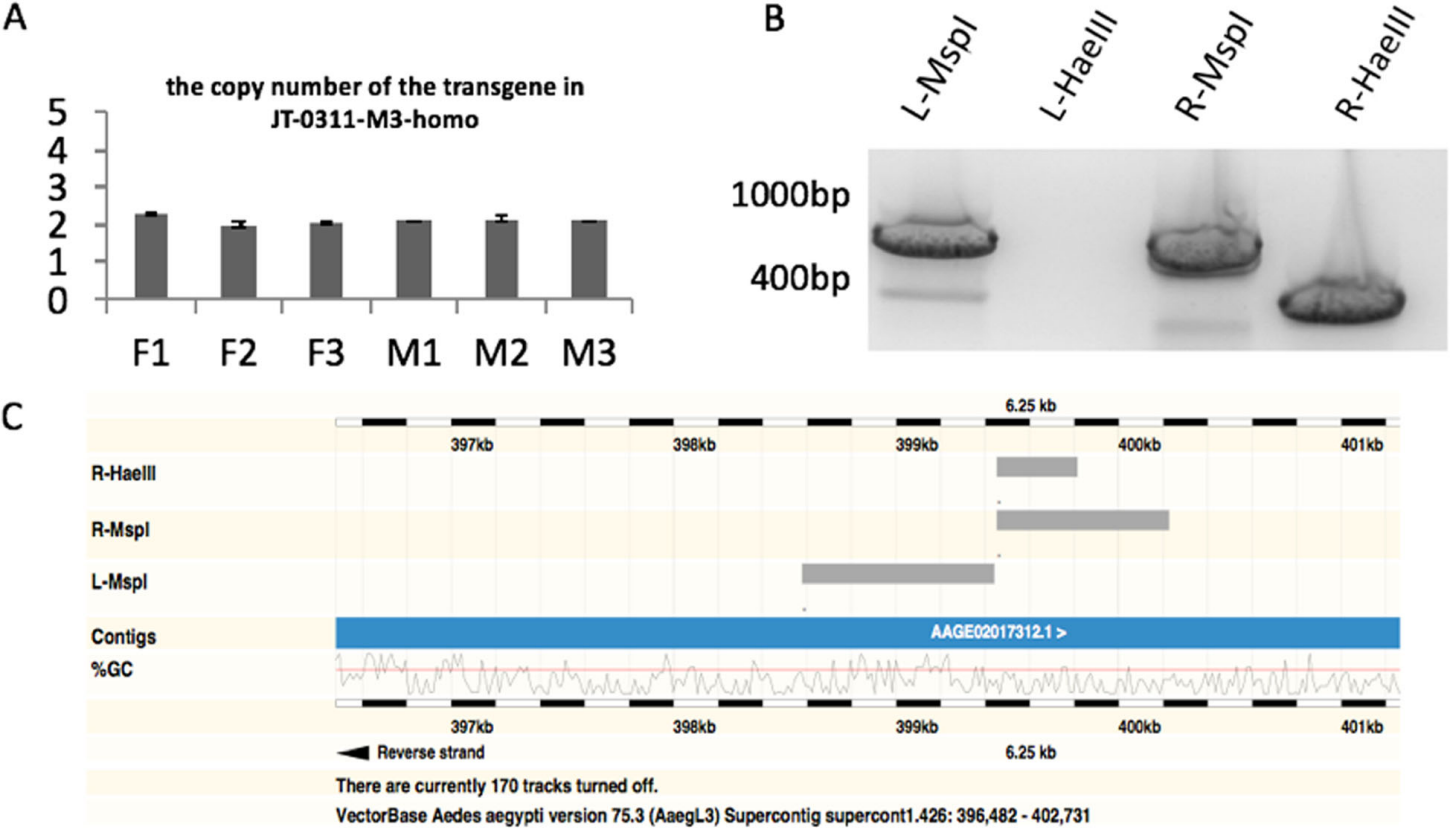
Transgene copy number and insertion site of the JT0311-M3-homo line. **a** The copy number of the transgene was determined by ddPCR. Copy numbers were determined for three females (F1-F3) and three males (M1-M3). Two haploid copies in all six individuals from a homozygous line indicate a single insertion event. **b** Gel image of iPCR that was used to determine the sequence flanking the insertion site. **c** Alignment of iPCR sequences to the *Ae. aegypti* genome

Transgenic mosquitoes were created by co-injecting the transgene-containing donor plasmid and the transposase-containing helper plasmid into the pre-blastoderm embryos of *Ae. aegypti* Liverpool strain. The donor plasmid (Fig. 1a) contains a transformation marker, EGFP driven by the 3xP3 promoter, and a firefly luciferase coding cassette under the control of the 1 kb upstream sequence of KLC2. The transformation marker and the luciferase reporter cassette were flanked by *piggyBac* recognition sequence and were integrated into the mosquito genome in the presence of the *piggyBac* transposase provided by the helper plasmid. Seven transgenic lines were obtained from a single injection. All lines had characteristic EGFP expression in the eye with slightly different signal strength and expression patterns (Fig. 1b, Additional file 1). To eliminate the expression variance due to segregation of the heterozygous transgene in the progeny, we chose 1 of the 7 lines, JT0311-M3, to generate homozygous line after multiple generations of crossing and screening (thereafter refers to JT0311-M3-homo line). The Droplet Digital PCR (ddPCR) [27] confirmed that there was only one copy of the transgene in the JT0311-M3-homo genome (Fig. 2a) and the inverse PCR (iPCR) detected that the transgenes were reversely inserted in the intergenic region between AAEL009760 and AAEL009761 in the Supercont1.426 with at least 200 kb away from the neighboring genes (Fig. 2b, c).

### The KLC2 promoter drives early zygotic expression of the luciferase transgene

**Fig. 3:**
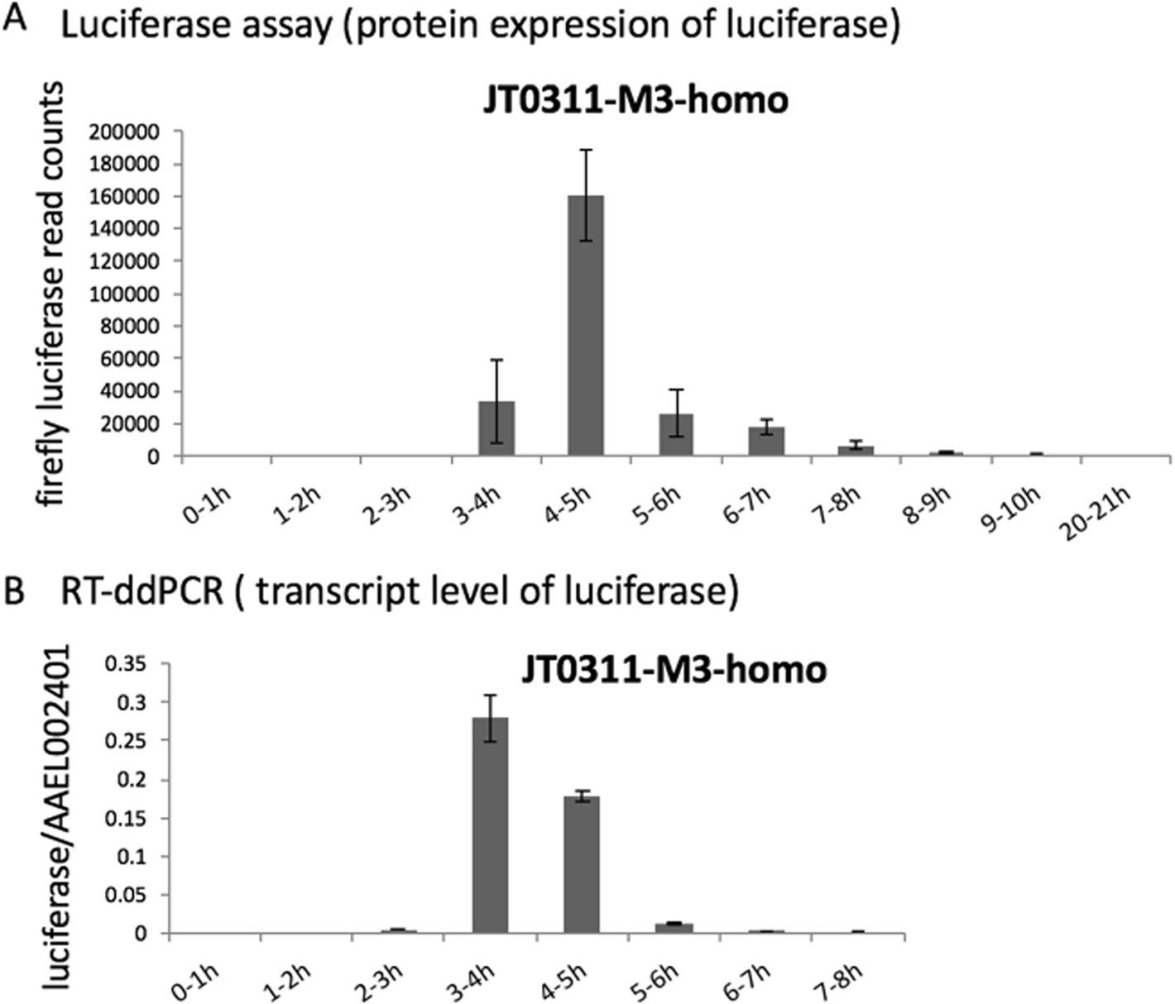
Profiles of the luciferase protein activity (**a**) and transcript level (**b**) in the early embryos of the JT0311-M3-homo line. **a** The luciferase assay showed that the luciferase signal was first observed in 3-4 h embryos, peaked in the 4-5 h embryos and disappeared after 9-10 h. **b** The RT-ddPCR showed that the luciferase transcript was observed in 2-3 h embryos, peaked in 3-4 h embryos and disappeared after 7-8 h. AAEL002401 was used as an internal reference

We tested the KLC2 promoter activity by monitoring luciferase expression in different developmental stages. Consistent with the transcription profile of the endogenous KLC2 gene in early embryos [28], transient luciferase signals were observed in eggs from 3-5 h post-egg laying (PEL) to 7-9 h PEL (Additional file 2) in all 7 lines. A more detailed luciferase assay was performed using JT0311-M3-homo early embryos with 1-hour interval (Fig. 3a). A reverse transcriptase ddPCR (RT-ddPCR) was conducted to profile the transcripts in early embryos (Fig. 3b). The luciferase assay results, which illustrate luciferase protein abundance, and the RT-ddPCR results, which illustrate luciferase transcripts abundance, showed good correlations in early embryos (Fig. 3a, b). Agreeing with the KLC2 expression profile [28], the luciferase gene was purely zygotic with no expression in 0-1 h and 1-2 h embryo. The transcription of luciferase gene started from 2-3 h PEL, peaked at 3-4 h PEL and dropped to background level after 6 h PEL. The measurement of luciferase protein showed approximate 1 h lag comparing with the transcripts, which started from 3-4 h embryos, peaked in 4-5 h embryos and disappeared in embryos older than 9 h. The expression profile of luciferase gene perfectly mimicked the expression profile of endogenous KLC2 in early embryos (Biedler & Tu, 2010,) (Additional file 3), indicating that the 1 kb upstream sequence of KLC2 was able to drive full expression in early embryos of *Ae. aegypti*.

### The KLC2 promoter also drives the expression of the transgene in testis

**Fig. 4:**
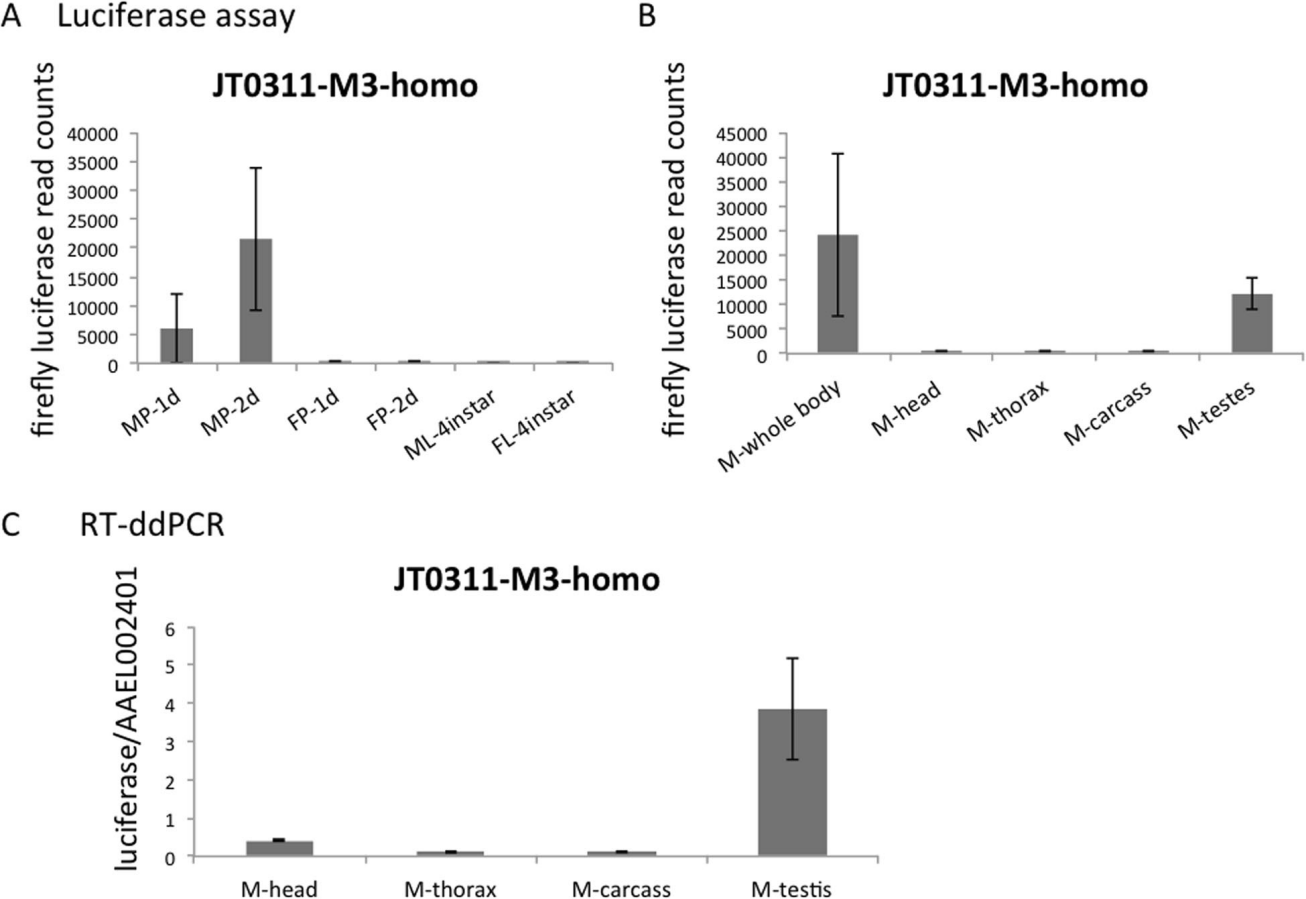
Testis expression of the transgene. **a** The luciferase protein was detected in 1 and 2 day old male pupae (MP-1d, MP-2d) but not in 1 and 2 day old female pupae (FP-1d, FP-2d), male larvae (ML-4instar), or female larvae (FL-4instar), according to luciferase assay. **b** The luciferase protein activity in adult male was mainly detected in the testes. **c** RT-ddPCR showed that the luciferase transcripts were mainly detected in the male testes

**Fig. 5:**
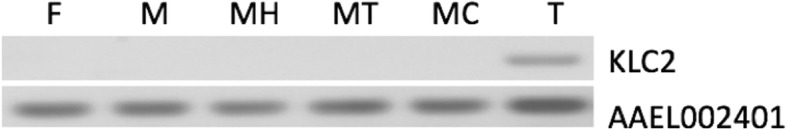
RT-PCR showed that the endogenous KLC2 gene was expressed in testes. Lane F: female whole body; Lane M: male whole body; Lane MH: male head; Lane MT: male thorax; Lane MC: male carcass (abdomen without testis); Lane T: testis. AAEL002401 was used as the internal reference

While the expression of luciferase gene was predominant in early embryos, luciferase assays showed additional expression peaks in pupae and adult males in six of the seven heterozygous lines (Additional file 2). Because these seven lines were from different G_0_ individuals and had slightly different EGFP patterns (Additional file 1), it is unlikely that they all had the same insertion site. All six lines had various levels of luciferase expression in pupae and adult males, indicating that this activity was not due to location effects. We therefore looked more carefully into the non-early-zygotic expression of luciferase in the JT0311-M3-homo line. Luciferase assays performed on sexed pupae showed that, like in the adult stage, the transgene was expressed only in male pupae (Fig. 4a). In the adult males, the luciferase signal was mainly from testes, as shown by the luciferase assay and RT-ddPCR (Fig. 4b, c). The male testis expression of the luciferase gene was unexpected because the endogenous KLC2 gene did not show male expression in previous RT-PCR or RNAseq analysis [28, 29] (Additional file 4). Nevertheless, when we did RT-PCR of the endogenous KLC2 on dissected male tissues, a band was observed in testis whereas no band could be detected when using male whole body as PCR template (Fig. 5).

## Discussion

Comparing with model organisms such as *Drosophila melanogaster*, the knowledge and application of functional genetic elements in the yellow fever mosquito *Ae. aegypti* are still quite limited. Often a tissue or temporal specific gene and its regulatory region were characterized first in *Drosophila*, and then their homologous counterparts were found and tested in mosquitoes [17, 20, 22, 30]. This transfer of knowledge does not always work given more than 200 million years of divergence between the two taxa. For example, the maternal and early zygotic promoters used to build the synthetic MEDEA element in *Drosophila* were derived from *Drosophila* genes *bicoid* and *bottleneck* that are not found in mosquitoes. The availability of the draft *Ae. aegypti* genome and the rapid development of next generation sequencing greatly facilitated the discovery of *Ae. aegypti* gene regulatory elements *de novo*. By applying transcriptome profiling, it is relative straightforward to find genes with expression in particular developmental stages or tissues. For example, Akbari et al. identified four germline promoters in *Ae. aegypti* through RNAseq recently [23]. In *Anopheles stephensi*, both maternal promoters and zygotic promoters have been tested using transgenic lines [31–33].

We previously found that KLC2 was a pure early zygotic gene with no maternal deposition and it is transcribed at the onset of the zygotic genome activation in *Ae. Aegypti* [28]. We chose the promoter of KLC2 for transgenic test based on a few observations. First, the expression of KLC2 was relatively high in early zygotic stage (ranked 13^th^ when sorted RPKM reads of all transcripts by ascending order in 0-2 h embryos and descending order in 2-4 h embryos). Secondly, the expression profile of KLC2 was clean and only in early zygotic stage according to RNAseq. Thirdly, unlike other top-ranked early zygotic genes, KLC2 has fewer closely related paralogues and is relatively easy to study. Fourthly, transient luciferase assay demonstrated that the 1 kb upstream region of KLC2 was capable of driving gene expression in 5-6 h embryos [25, 28]. In this study, we showed that the 1 kb promoter of KLC2 faithfully drove gene expression in transgenic *Ae. aegypti* early embryos. The transcription profile of the transgene in embryos was the same as that of endogenous KLC2 gene.

We also noticed a clear transgene expression in male pupae and adult testes. This male-specific activity was unlikely due to location of the transgene insertion sites because multiple lines bore the same expression pattern. Indeed, when we aligned the RNAseq data from two independent studies to the neighboring region of JT0311-M3-homo insertion site, we found no male-specific transcribed region [28, 29] (Additional file 5). The luciferase signal was barely detected in the 4^th^ instar male larvae, when spermatogonial mitosis begins to be observed [34]. This signal started to increase in young male pupae, when primary and secondary spermatocytes are generated through meiosis. The signal was highest in old male pupae and male adults, when most steps of spermatogenesis have completed [34]. It is interesting that in mouse, while the two major KLC genes, KLC1 and KLC2 were not expressed in mature spermatids, another KLC gene, KLC3 was primarily expressed in post-meiotic male germ cells and was involved in sperm tail midpiece formation and sperm function [35, 36]. RT-PCR of dissected tissues using primers specific for *Ae. aegypti* KLC2 confirmed the expression of the KLC2 gene in adult testes. Taking together, our results suggest that the KLC2 may play a role in spermatogenesis or other testis-related functions in *Ae. aegypti*. However, functional analysis is needed to determine the precise role of KLC2 in male development.

We noticed that the testis transcription of the endogenous KLC2 was very weak while the expression of transgene was relatively high in testes. There could be another inhibiting element outside the 1 kb promoter region that suppresses testis expression of KLC2. Another explanation is that the stability of luciferase transcript and protein are higher than that of endogenous KLC2. The male expression of KLC2 was not detected in our RNAseq data [25, 28]. This might be because we sequenced male whole bodies and the weak expression of KLC2 in testes was overwhelmed by transcripts in other parts of males. Indeed, RT-PCR did not show clear expression of KLC2 when male whole body was used. Curiously, RNAseq of 4 d male testes and accessory gland also failed to detect reads of KLC2 in an earlier study [29]. It is possible that the sequencing depth was not high enough to recover low abundant transcripts, or like in the early embryos, KLC2 may be transiently expressed in the testes, which could be missed during sample collection. Either scenario indicates the limitation of RNAseq in profiling genes that are transcribed in a narrow time window or in a small number of cells. Increasing sequencing depth and sample coverage will mitigate this problem.

## Conclusions

The 1 kb promoter region of the KLC2 gene is able to drive gene expression at the beginning of the syncytial blastoderm stage in *Ae. aegypti*, making it a good resource for studying gene function during embryonic development and potentially for delivering transgene products to early embryos. For example, ectopic expression or knockdown of genes involved in sex-determination during early embryonic stages could produce genetic sexing strains, which are useful for population suppression approaches like the sterile insect technique [14–16]. Moreover, our transgenic reporter assays showed that cautions are needed when interpreting RNA sequencing data because transient and/or tissue-specific transcription may go undetected even in relatively broadly sampled datasets.

## Methods

### Mosquitoes

*Aedes aegypti* Liverpool strain (wild type) and all transgenic mosquitoes were reared at 28°C and 60% relative humidity on a 16 hr light/8 hr dark photoperiod. Mosquito preblastoderm embryo injection and G_0_ transgenic mosquito screening were performed by the Insect Transformation Facility in Institute for Bioscience and Biotechnology Research at the University of Maryland (http://www.ibbr.umd.edu/facilities/itf). Microinjection into *Ae. aegypti* embryos was performed according to standard protocols [37] and the injection solution contains a 150 ng/μl donor plasmid (Fig. 1a, Additional file 6), a 300 ng/μl *piggyBac* transposase (phsp-Pbac) helper plasmid [38]. Third- or fourth-instar larvae of the transgenic lines were screened under fluorescent microscope for EGFP signal. All heterozygous lines were maintained by crossing transgenic positive males with wild type virgin females. To generate a homozygous line, heterozygous transgenic males and females were crossed. The positive offspring were then crossed with each other. When it was time to lay eggs, 20 females were allowed to lay eggs in individual tubes. The eggs were hatched separately, and larvae were inspected for any negatives. Any batches of larvae that appeared all positive were allowed to interbreed and the individual female egg collection was repeated. After several generations of inbreeding, the line was assumed homozygous when no negative larvae were observed.

### Copy number determination

To determine the copy number of the transgene in the mosquito genome, ddPCR was performed on genomic DNA (gDNA) of JT0311-M3-homo mosquitoes using Bio-rad QX100 Droplet Digital PCR system following the previously published method [27]. Genomic DNAs were extracted from 3 adult male individuals and 3 adult female individuals by ZYMO Quick-gDNA Miniprep kit. Approximately 300-500 ng gDNA were digested with *Dpn*I at 37 °C for 1 h and used as ddPCR template after ethanol precipitation. *Dpn*I restriction digestion was performed to ensure separation of potential tandem repeats. A known single copy gene, nk homeobox protein AAEL006597, was used as an internal control. In all 6 tested-mosquitoes, two haploid copies were detected (Fig. 2a). Because JT0311-M3-homo is a homozygous line for transgene, this result indicated that there was a single insertion of the transgene. All primers and probes and ddPCR conditions are described in Additional file 2.

### Inverse PCR

Approximately 1 μg of JT0311-M3-homo gDNA was digested with either MspI or HaeIII. After digestion, the product was ethanol precipitated and subjected to overnight ligation using T4 ligase at 4 °C. The ligation product was again ethanol precipitated and used for PCR. Primers were designed to amplify either the left arm of the transgene plus genomic flanking sequences (primer set L1 and L2, Fig. 1a) or the right arm of transgene plus genomic flanking sequences (primer set R1 and R2, Fig. 1a). PCR products could be amplified from three of the four combinations (Fig. 2b). The bands were gel purified and sent for sequencing. The sequencing results were BLAST against *Ae. aegypti* genome. The BLAST output from left arm and right arm were compared to pin point the insertion site. Segments in supercont1.426 were the best hit in all three combinations, with left-arm flanking sequences aligning to supercont1.426: 398577–399439 and right-arm flanking sequences aligning to supercont1.426: 399453–399813 (*Hae*III) or 400224 (*Msp*I) (Fig. 2c). This location was the only location that showed a significant match in the BLAST output of all three inverse PCR sequences, confirming that there was a single insertion event. Primers and iPCR conditions are described in Additional file 2 and the approximate positions of the primers relative to the transformation donor plasmid are illustrated in Fig. 1.

### Luciferase assays

Firefly luciferase assays were performed using reagents from Promega. Briefly, mosquito embryos or tissue samples were grounded in 50 μl Passive Lysis Buffer. Twenty microliters of lysate were added into luciferase reagent (1× luciferase substrate in buffer) and measured for luminescence immediately in GloMax 20/20 luminometer. We used 10 embryos, 1 larva, 1 pupa, 1 adult male or female whole body, 2 heads, 2 thoraces, 2 carcasses (abdomen without testes) or 2 pairs of testes for each measurement. The sex of pupae was differentiated by morphological structure [39]. The sex of larvae was decided by PCR of a male specific gene named myosex [40] on the gDNA extracted from a small amount of larvae tissue using a housekeeping gene (AAEL006597) as quality control. Primers and PCR conditions are described in Additional file 2.

### Reverse transcriptase PCR

Total RNAs were extracted from staged embryos, adult males, adult females, male heads, thoraxes, carcass, or testes by ZYMO Quick-RNA MiniPrep kit. Approximately 20 ng RNAs were reverse transcribed to cDNA by Invitrogen SuperScript III RT kit. Regular RT-PCR and RT-ddPCR were performed on different cDNA samples using FAM-labeled probes targeting luciferase gene or endogenous KLC2 gene and Hex-labeled probe targeting an internal control housekeeping gene AAEL002401. The concentrations of target genes were normalized by dividing the concentration of AAEL002401 in each PCR well. All primers and probes and RT-PCR and RT-ddPCR conditions are described in Additional file 2.

## Additional files

Additional file 1: Marker phenotypes of the seven transgenic lines. (XLSX 27 kb)

Additional file 2: Luciferase expression in seven independent transgenic lines. Firefly luciferase assays showed that except JT0311-F2, other transgenic lines all have two expression peaks during the mosquito life-cycle. The first one is in early embryos and the second one is in pupa or adult males. (PNG 99 kb)

Additional file 3: Primers and probes used in this study. (DOCX 19 kb)

Additional file 4: Expression profile of KLC2 (AAEL011410) based on RNA sequencing data adopted from (a) Biedler et al. [25] and (b) Akbari et al. [29]. Both RNAseq data showed pure early zygotic expression of KLC2 without expression in adult males or male testes. (PNG 95 kb)

Additional file 5: Alignment of RNAseq data [29] to the genomic region surrounding the insertion site. The grey bars in the top panel indicate the alignment of iPCR sequence to the genome, which enabled the identification of the insertion site. The mapping results of various RNAseq data showed that the genes in the surrounding region were not transcribed in only males. Thus, the observed testis-biased expression is unlikely caused by enhancers or promoters in the neighboring region. (PNG 93 kb)

Additional file 6: The sequence of the luciferase reporter cassette. (DOCX 116 kb)

## Abbreviations

PEL: Post-egg laying
iPCR: Inverse PCR
ddPCR: Droplet digital PCR
