# Supplementary figures and images for: Functional analysis of the promoter of an early zygotic gene KLC2 in Aedes aegypti
Source: Parasit Vectors. 2018 Dec 24;11(Suppl 2):655. doi: 10.1186/s13071-018-3210-0 (PMC6305062; doi:10.1186/s13071-018-3210-0)

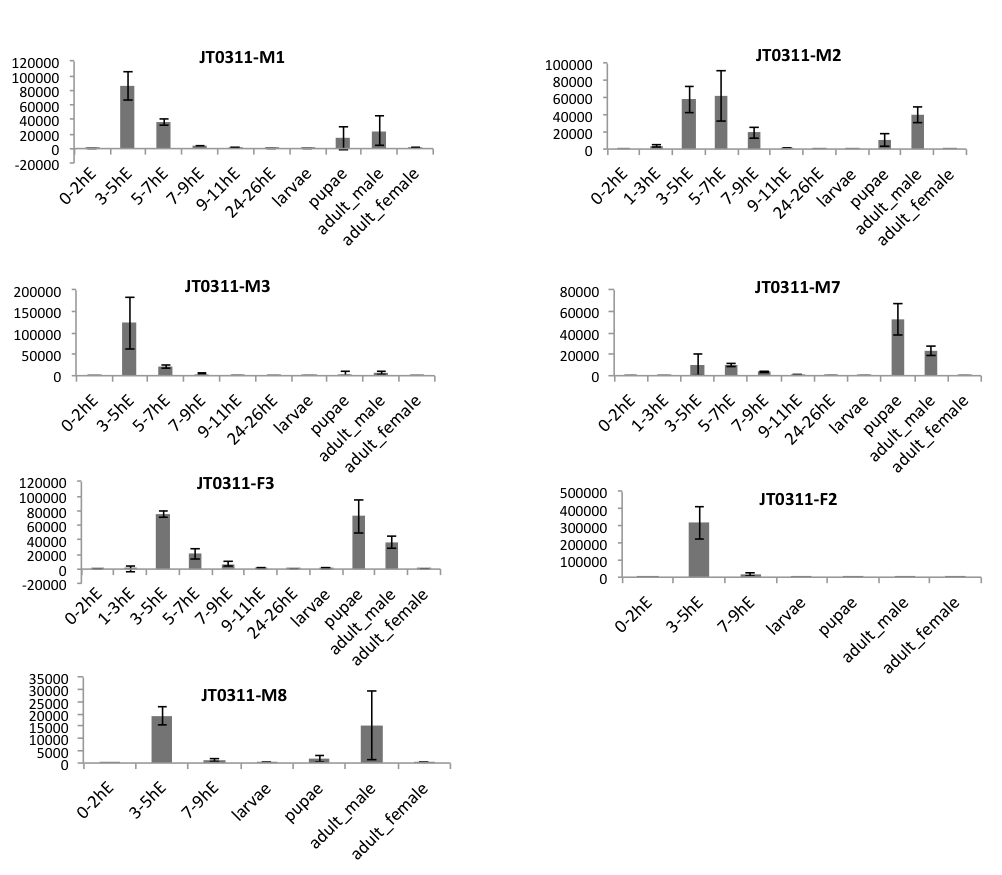

Supplement: Supplementary file 2 — Luciferase expression in seven independent transgenic lines. Firefly luciferase assays showed that except JT0311-F2, other transgenic lines all have two expression peaks during the mosquito life-cycle. The first one is in early embryos and the second one is in pupa or adult males. (PNG 99 kb) [file 13071_2018_3210_MOESM2_ESM.png]

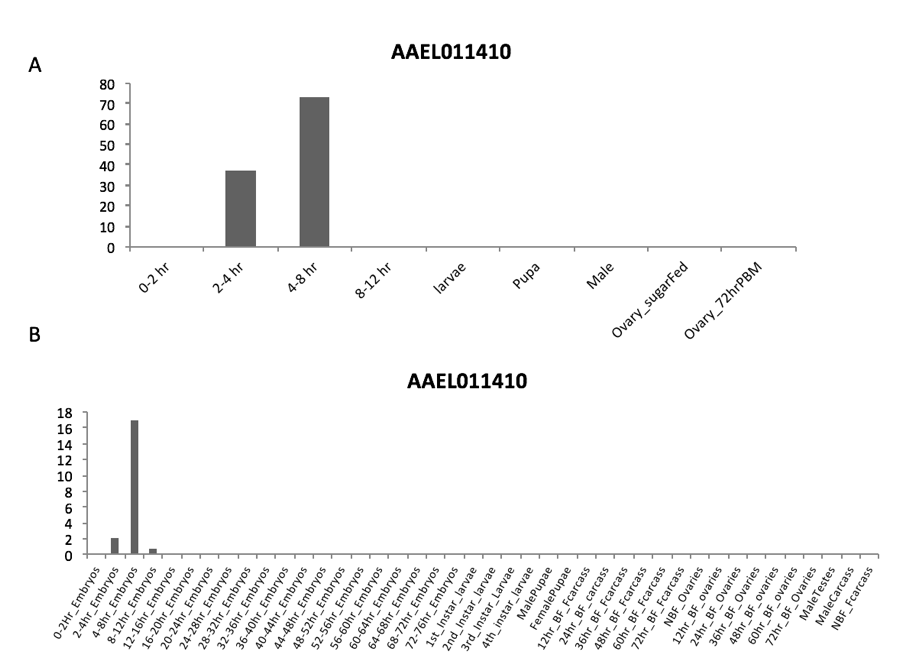

Supplement: Supplementary file 4 — Expression profile of KLC2 (AAEL011410) based on RNA sequencing data adopted from (a) Biedler et al. [25] and (b) Akbari et al. [29]. Both RNAseq data showed pure early zygotic expression of KLC2 without expression in adult males or male testes. (PNG 95 kb) [file 13071_2018_3210_MOESM4_ESM.png]

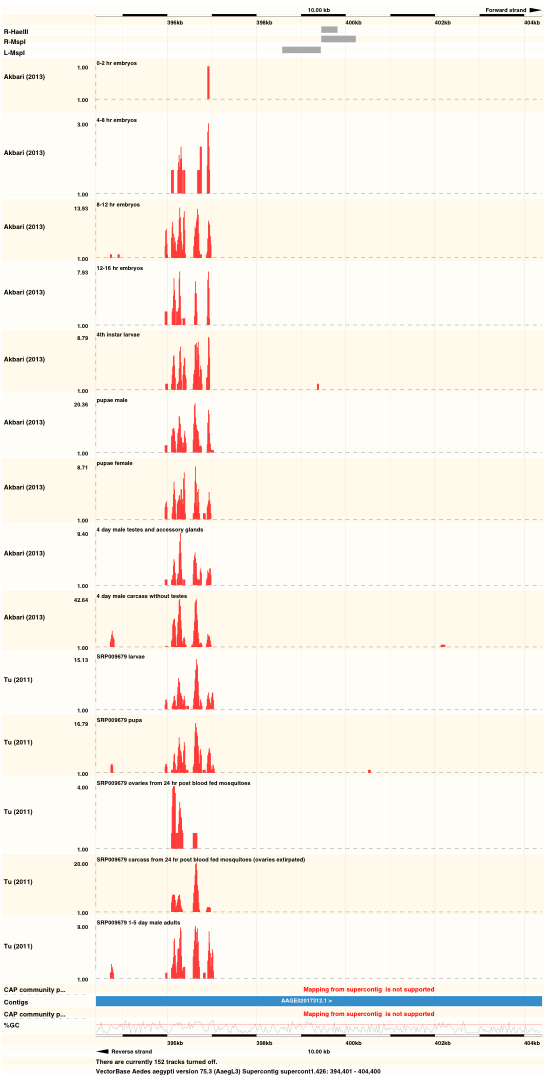

Supplement: Supplementary file 5 — Alignment of RNAseq data [29] to the genomic region surrounding the insertion site. The grey bars in the top panel indicate the alignment of iPCR sequence to the genome, which enabled the identification of the insertion site. The mapping results of various RNAseq data showed that the genes in the surrounding region were not transcribed in only males. Thus, the observed testis-biased expression is unlikely caused by enhancers or promoters in the neighboring region. (PNG 93 kb) [file 13071_2018_3210_MOESM5_ESM.png]
