# Supplementary material for: Functional analysis of the promoter of an early zygotic gene KLC2 in Aedes aegypti
Source: Parasit Vectors. 2018 Dec 24;11(Suppl 2):655. doi: 10.1186/s13071-018-3210-0 (PMC6305062; doi:10.1186/s13071-018-3210-0)
Supplement: Supplementary file 3 — Primers and probes used in this study. (DOCX 19 kb) [file 13071_2018_3210_MOESM3_ESM.docx]

| Luciferase-F^1^ | TTGGCAGAAGCTATGAAACG |
| --- | --- |
| Luciferase-R^1^ | GCAACTGCAACTCCGATAAA |
| Luciferase probe^1^ | 5’FAM-CAATTCTTTATGCCGGTGTTGG-3’BHQ-1 |
| AAEL006957-F^2^ | CGTGGTGCAGATAGTGAACG |
| AAEL006957-R^2^ | CATGTTAAGTTTGCCATAAAATTCG |
| AAEL006957 probe^2^ | 5’Hex-TGGTGACTTGGGAAGGATGAAGTA-3’BHQ-1 |
| AAEL002401-F^3^ | TGGCCAGATAGTCGATGTAAT |
| AAEL002401-R^3^ | TACAAGATGCGCAATGGATA |
| AAEL002401 probe^3^ | 5’-Hex-CGTATTGGTTGGAGGCTATGACGA-3’BHQ-1 |
| iPCR_pBAC_L1^4^ | TGGCTCTTCAGTACTGTCAT |
| iPCR_pBAC_L2^4^ | TACGCATGATTATCTTTAACGTA |
| iPCR_pBAC_L1nest^5^ | CACTTCATTTGGCAAAATAT |
| iPCR_pBAC_L2nest^5^ | GTCACAATATGATTATCTTTCTAGG |
| iPCR_pBAC_R1^6^ | GACACTTACCGCATTGACAAG |
| iPCR_pBAC_R2^6^ | CAAAGTCCACGAGGCGTAG |
| iPCR_pBAC_R1nest^7^ | ACTGAGATGTCCTAAATGCACAG |
| iPCR_pBAC_R2nest^7^ | CCGAGTCTCTGCACTGAAC |
| Myo-sex-F^8^ | CCTTCAAGCACACCGTTACA |
| Myo-sex-R^8^ | TCACTATGCAGGAGTTGTTTCG |
| KLC2-F2^9^ | CTACCAGAAGCCATCAAACAT |
| KLC2-R2^9^ | GATTCACTGTCACCATTCTGTGT |

**Additional file 3:** Primers and probes used in this study

1. Digital droplet PCR (ddPCR) primer and probe set used to determine the copy number of the luciferase transgene. PCR cycling condition: 95 ℃ for 10 minutes, then (94 ℃ 30 seconds and then 58 ℃ 1 minute) x 40 cycles. The same set of primers and probe have also been used to quantify the luciferase transcript levels in the transgenic line by performing RT-ddPCR. The PCR cycling condition is the same as what was used for copy number analysis. A ddPCR assay for a copy number reference is described in 2. A RT-ddPCR assay for an internal reference for normalization is described in 3.
2. ddPCR primer and probe set for a known single copy gene AAEL006957, which is used as a copy number reference for comparison with the luciferase transgene. PCR cycling condition: the same as described in 1. The same primers have also been used as positive controls for regular PCR without the probe.
3. Digital droplet PCR (ddPCR) primer and probe set used for quantifying the transcript level of an *Ae. aegypti* gene (AAEL002401), which is used for normalization. PCR cycling condition: the same as described in 1. The same primers have also been used as positive controls for regular RT-PCR without the probe.
4. Inverse PCR primers used to amplify genomic regions flanking the left arm of the piggyback insertion as illustrated in Figure 1. PCR cycling condition: 95 ℃ for 3 minutes, (95 ℃ 30 seconds, 55 ℃ 30 seconds, 72 ℃ 1 minute) x 32 cycles.
5. Nested inverse PCR primers used to amplify genomic regions flanking the left arm of the piggyback insertion. The template used for this nested PCR is the PCR product from the above inverse PCR reaction PCR cycling condition: the same as described in 4.
6. Inverse PCR primers used to amplify genomic regions flanking the right arm of the piggyback insertion as illustrated in Figure 1. PCR cycling condition: the same as described in 4.
7. Nested inverse PCR primers used to amplify genomic regions flanking the right arm of the piggyback insertion. The template used for this nested PCR is the PCR product from the above inverse PCR reaction. PCR cycling condition: the same as described in 4.
8. Primers for gDNA amplification of a male-specific gene in the male-determining locus[[40]]. This PCR reaction is used to determine the sex of the mosquito larvae or pupae. PCR cycling condition: similar to what is described in 4 but the annealing temperature is 63 ℃
9. RT-PCR primers used to detect KLC2 transcription. PCR cycling condition: similar to what is described in 4 but the annealing temperature is 56 ℃
