# Supplementary material for: Functional analysis of the promoter of an early zygotic gene KLC2 in Aedes aegypti
Source: Parasit Vectors. 2018 Dec 24;11(Suppl 2):655. doi: 10.1186/s13071-018-3210-0 (PMC6305062; doi:10.1186/s13071-018-3210-0)
Supplement: Supplementary file 6 — The sequence of the luciferase reporter cassette. (DOCX 116 kb) [file 13071_2018_3210_MOESM6_ESM.docx]

**Additional file 6:**

The sequence of the luciferase reporter cassette is shown below.

>KLC2Promoter-Luc_(KLC2 promoter plus 5’ UTR: 1-1105 bp, Luciferase ORF: 1106-3011 bp, Start and Stop codons are in bold)

CGACTGTGGCCAACTTCAAGCCACCAGGGTGCATCATACCGCACCACACCAGTGGACGAACATGCCTCATGTTGAGGCCACTTACACACCACAAATCCTGGCAGCACTTTACTCACCTGAAATAATTCGTCATACACTCATTCATGTTGCATATCTAGAAACATAAAACAATTAGTATCAAATCGTTAAAACACAAAGTAATCAAATTACCCTAAAATATACTGCTCCAATAACCGGGTTCTAGGATACTTCATACAGACACAATATAACTATCAACAAATGCAATAATCGATCATTGATTGCTTTTTGATACTTGCGTTACTCTGTCTGCACAGAAATTGCTGCAATTTATGGCCACCTATCCAAATATGGTAAATGATAAGAAATTGTATTGAATTAGATAATATTTATGGATTTCTATTTGATGGAGGATGAGAAGATCTGACAAGCCATTTGGAATGTCTTTCATTTCCTTGCATTCCTCAGCACGCTGATCGTTCTCTCTGTTACGGTAAAATGCAGTTGGTCCACTCAGAGTGCATAATTTTATCGTTCATCTAAATTTGTTGCTAAATATTTCTCGCAGTTAACAGCATTGATCAAATCTGAGCAATTGAAAAGGAAGCTAAGAAAATTGCACCAGAAGATGGAATCATCCTTTTTTTTAAATTTTGTACTTGGTCCGCTCTGACTTATCGCCGACCACATTGTGTTTCGTAAAATATGGTGATATCAAGTCTACCCATTCTCCCCTTTAGCTAAATAGCTTTAAACATAATTTTCATAACACCATCCTTTGAATATAGTGAGACGTAAATTTGTAGAGGTAGCAAATCGTGCCACTTAATGTTGCCTTTGAGTTTACCTGTTCAGCACGCGTCTTTTAGTTGGCCAGTAAATTAATAATTTGTTACTGTATACCTATAAGAGGGTAGCCGGTAATGGGTACATATCATTTCGATCGTTTGACTATTTATAGTGTATCAAAATCAAGTGCATTTCAGTTACTTCAACTAGTTTTCCAAATCTTCCAATCAATCAACCCCGGGCTCGAGATCTGCGATCTAAGTAAGCTTGGCATTCCGGTACTGTTGGTAAAGCCACC**ATG**GAAGACGCCAAAAACATAAAGAAAGGCCCGGCGCCATTCTATCCGCTGGAAGATGGAACCGCTGGAGAGCAACTGCATAAGGCTATGAAGAGATACGCCCTGGTTCCTGGAACAATTGCTTTTACAGATGCACATATCGAGGTGGACATCACTTACGCTGAGTACTTCGAAATGTCCGTTCGGTTGGCAGAAGCTATGAAACGATATGGGCTGAATACAAATCACAGAATCGTCGTATGCAGTGAAAACTCTCTTCAATTCTTTATGCCGGTGTTGGGCGCGTTATTTATCGGAGTTGCAGTTGCGCCCGCGAACGACATTTATAATGAACGTGAATTGCTCAACAGTATGGGCATTTCGCAGCCTACCGTGGTGTTCGTTTCCAAAAAGGGGTTGCAAAAAATTTTGAACGTGCAAAAAAAGCTCCCAATCATCCAAAAAATTATTATCATGGATTCTAAAACGGATTACCAGGGATTTCAGTCGATGTACACGTTCGTCACATCTCATCTACCTCCCGGTTTTAATGAATACGATTTTGTGCCAGAGTCCTTCGATAGGGACAAGACAATTGCACTGATCATGAACTCCTCTGGATCTACTGGTCTGCCTAAAGGTGTCGCTCTGCCTCATAGAACTGCCTGCGTGAGATTCTCGCATGCCAGAGATCCTATTTTTGGCAATCAAATCATTCCGGATACTGCGATTTTAAGTGTTGTTCCATTCCATCACGGTTTTGGAATGTTTACTACACTCGGATATTTGATATGTGGATTTCGAGTCGTCTTAATGTATAGATTTGAAGAAGAGCTGTTTCTGAGGAGCCTTCAGGATTACAAGATTCAAAGTGCGCTGCTGGTGCCAACCCTATTCTCCTTCTTCGCCAAAAGCACTCTGATTGACAAATACGATTTATCTAATTTACACGAAATTGCTTCTGGTGGCGCTCCCCTCTCTAAGGAAGTCGGGGAAGCGGTTGCCAAGAGGTTCCATCTGCCAGGTATCAGGCAAGGATATGGGCTCACTGAGACTACATCAGCTATTCTGATTACACCCGAGGGGGATGATAAACCGGGCGCGGTCGGTAAAGTTGTTCCATTTTTTGAAGCGAAGGTTGTGGATCTGGATACCGGGAAAACGCTGGGCGTTAATCAAAGAGGCGAACTGTGTGTGAGAGGTCCTATGATTATGTCCGGTTATGTAAACAATCCGGAAGCGACCAACGCCTTGATTGACAAGGATGGATGGCTACATTCTGGAGACATAGCTTACTGGGACGAAGACGAACACTTCTTCATCGTTGACCGCCTGAAGTCTCTGATTAAGTACAAAGGCTATCAGGTGGCTCCCGCTGAATTGGAATCCATCTTGCTCCAACACCCCAACATCTTCGACGCAGGTGTCGCAGGTCTTCCCGACGATGACGCCGGTGAACTTCCCGCCGCCGTTGTTGTTTTGGAGCACGGAAAGACGATGACGGAAAAAGAGATCGTGGATTACGTCGCCAGTCAAGTAACAACCGCGAAAAAGTTGCGCGGAGGAGTTGTGTTTGTGGACGAAGTACCGAAAGGTCTTACCGGAAAACTCGACGCAAGAAAAATCAGAGAGATCCTCATAAAGGCCAAGAAGGGCGGAAAGATCGCCGTG**TAA**TTCTAGAGTCGGGGCGGCCGGCCGCTTCGAGCAGACATGATAAGATACATTGATGAGTTTGGACAAACCACAACTAGAATGCAGTGAAAAAAATGCTTTATTTGTGAAATTTGTGATGCTATTGCTTTATTTGTAACCATTATAAGCTGCAATAAACAAGTTAACAACAACAATTGCATTCATTTTATGTTTCAGGTTCAGGGGGAGGTGTGGGAGGTTTTTTAAAGCAAGTAAAACCTCTACAAATGTGGTA
